# Initial experience on abdominal photon-counting computed tomography in clinical routine: general image quality and dose exposure

**DOI:** 10.1007/s00330-022-09278-1

**Published:** 2022-12-08

**Authors:** Benjamin V. Becker, Hanns Leonhard Kaatsch, Kai Nestler, Daniel Overhoff, Julian Schneider, Daniel Dillinger, Joel Piechotka, Marc A. Brockmann, Reinhard Ullmann, Matthias Port, Harry Scherthan, Stephan Waldeck

**Affiliations:** 1 Department of Radiology and Neuroradiology, Bundeswehr Central Hospital Koblenz, Rübenacher Straße 170, 56072 Koblenz, Germany; 2 Bundeswehr Institute of Radiobiology affiliated to Ulm University, Neuherbergstrasse 11, 80937 Munich, Germany; 3 Department of Radiology and Nuclear Medicine, University Medical Center Mannheim, Theodor-Kutzer-Ufer 1-3, 68167 Mannheim, Germany; 4 Department of Vascular and Endovascular Surgery, Bundeswehr Central Hospital Koblenz, Rübenacher Straße 170, 56072 Koblenz, Germany; 5 Department of Neuroradiology, University Medical Center Mainz, Langenbeckstrasse 1, 55101 Mainz, Germany

**Keywords:** Tomography, Dose reduction, Signal-to-noise ratio, DNA damage

## Abstract

**Objectives:**

Photon-counting computed tomography has lately found its way into clinical routine. The new technique could offer substantial improvements regarding general image quality, image noise, and radiation dose reduction. This study evaluated the first abdominal examinations in clinical routine and compared the results to conventional computed tomography.

**Methods:**

In this single-center retrospective study, 66 patients underwent photon-counting and conventional abdominal CT. Four radiologists assessed general image quality, image noise, and image artifacts. Signal-to-noise ratio and dose properties of both techniques within the clinical application were compared. An *ex vivo* phantom study revealed the radiobiological impact by means of DNA double-strand break foci in peripheral blood cells by enumerating γ-H2AX+53BP1 foci.

**Results:**

General image quality in accordance with the Likert scale was found superior for photon-counting CT (4.74 ± 0.46 vs. 4.25 ± 0.54; *p* < 0.001). Signal-to-noise ratio (*p* < 0.001) and also dose exposure were higher for photon-counting CT (DLP: 419.2 ± 162.2 vs. 372.3 ± 236.6 mGy*cm; *p* = 0.0435). CT exposure resulted in significantly increased DNA damage in comparison to sham control (*p* < 0.001). Investigation of the average foci per cell and radiation-induced foci numbers revealed significantly elevated numbers (*p* = 0.004 and *p* < 0.0001, respectively) after photon-counting CT.

**Conclusion:**

Photon-counting CT in abdominal examinations showed superior results regarding general image quality and signal-to-noise ratio in clinical routine. However, this seems to be traded for a significantly higher dose exposure and corresponding double-strand break frequency. Optimization of standard protocols in further clinical applications is required to find a compromise regarding picture quality and dose exposure.

**Key Points:**

*• Photon-counting computed tomography promises to enhance the diagnostic potential of medical imaging in clinical routine.*

*• Retrospective single-center study showed superior general image quality accompanied by higher dose exposure in initial abdominal PCCT protocols compared to state-of-the-art conventional CT.*

*• A simultaneous ex vivo phantom study revealed correspondingly increased frequencies of DNA double-strand breaks after PCCT.*

**Supplementary Information:**

The online version contains supplementary material available at 10.1007/s00330-022-09278-1.

## Introduction

Computed tomography (CT) is a widely used and essential tool in modern diagnostic radiology. Since its implementation in the 1970s, technological progress has led to increased diagnostic accuracy concomitant with a decrease in radiation dose exposure for single examinations [1–3]. In addition, applications utilizing dual-energy (DE) technique for tissue differentiation or reconstruction of virtual non-contrast images have further enhanced the diagnostic potential of CT in recent years [4, 5].

However, the current scanner technology still offers potential for improvement with regard to several aspects. Limited spatial resolution impairs the evaluation of small anatomical structures, such as tympanal ossicles, the pulmonary interstitium, or small vessels in CT angiography [6]. Furthermore, a higher spatial resolution could be beneficial for reducing blooming artifacts by calcified plaques in small vessels [7]. Despite iterative reconstruction algorithms, filtering techniques, and the use of integrated electronics in detectors, image quality in CT is still hampered by image noise, especially under special circumstances (e.g., obese patients, low-dose imaging/protocols) [8, 9].

Although technological advances have led to decreasing dose burdens of individual CT examinations in recent years [10], radiation exposure is a constant matter of concern in computed tomography as the overall number of performed examinations is still rising. For example, in the USA, numbers increased from 62 million in 2006 to 74 million in 2016, contrary to the trend of otherwise decreasing dose exposure [3]. In Germany, CT examinations are responsible for 68% of civilization-related dose exposure, although only accounting for 10% of performed examinations in 2018. Among those, abdominal CT represents the examination with the second-highest individual effective dose [10]. Notwithstanding, it can be assumed that the overall harm of modern CT diagnostics is rather small, radiation exposure due to these CT examinations has been shown to cause genotoxic stress and gene expression changes of so far unresolved biological relevance [11–15].

In the context of further technological development of CT, photon-counting computed tomography (PCCT) could be a major improvement to overcome the limitations mentioned above. In contrast to conventional detectors based on solid-state scintillators, PCCT detectors consist of a semiconductor layer with energy-resolving properties. This new technology enables a comparatively higher spatial resolution by omitting additional separation layers in detector cells and reducing image noise by eliminating low-energy x-rays representing electronic noise and backscattering based on their registered photon energy levels [16, 17]. Moreover, PCCT provides improved iodine contrast and low-dose imaging, resulting in less patient dose while maintaining adequate diagnostic quality [18–20].

Until recently, PCCT scanners were only available in research settings. With the FDA approval in September 2021, the first PCCT scanner was commercially available for clinical applications. However, based on the lack of experience regarding its performance in clinical routine, it remains unclear whether PCCT will become a standard tool in clinical diagnostic imaging. In this study, we therefore examined clinical routine abdominal PCCT examinations focusing on general image quality, image noise, image artifacts, dose exposure, and its impact on DNA integrity. We also compared the results to a conventional CT scanner and aimed to gain insights into the diagnostic advantages and limitations of PCCT in clinical routine.

## Methods

### Study population

All patients underwent single-phase (portal-venous) contrast-enhanced abdominal PCCT for medical reasons in clinical routine. Patients were retrospectively included during the first six weeks after installation (December 2021 to January 2022). For comparison, matched samples of patients undergoing conventional CT examinations during this time period were selected. The study is a single-center trial including a total of 66 different CT examinations of 65 patients. One patient was scanned with both devices during the examination period.

### CT examinations

Photon-counting CT was performed on a NAEOTOM Alpha scanner (Siemens Healthineers). Automated mAs modulation was used with a BQ 145 presetting, resulting in 145 mAs at 120 kV. Collimation was 144 rows × 0.4 mm with a rotation time of 0.5 s, a pitch of 0.8 and no additional hardware filter applied. For conventional CT examinations, a modern 64-row scanner was used (SOMATOM go.Top, Siemens Healthineers). Automated mAs modulation with 110 mAs at 120 kV was used. Collimation was 64 rows × 0.6 mm with a rotation time of 0.5 s, a pitch of 0.8 and no hardware filter applied. Images from both modalities were reconstructed in tri-plane views (1 mm axial soft-tissue kernel (Br40) with 1 mm increment; 3 mm coronal soft tissue kernel (Br40) with 1 mm increment and 3 mm sagittal bone kernel (Br60) with 3 mm increment). QIR (Siemens Healthineers) was used as the reconstruction algorithm.

Given dose-length product (DLP) and CT dose index (CTDI_w_body_) were evaluated. The effective dose for CT exposure was calculated according to the ICRP report 103 using Monte Carlo model-based dose estimation implemented in a dose management software tool (Radimetrics, Bayer). Furthermore, k-factor-based effective dose calculations with a conversion factor of 0.015 were performed in accordance with Shrimpton et al [21].

### Image evaluation

Images were evaluated individually by four experienced radiologists (E1-4, experience in abdominal CT 7–12 years). Radiologists were blinded to device and clinical details. Likert-Scales were provided regarding general image quality, image noise, and image artifacts. A score of 1 to 5 (best) could be chosen for each item, as seen in Table 1. Images were evaluated in DICOM format via JiveX (Version 5.1.0.20; Visus Health IT) under constant conditions. For further evaluation of general image quality, the signal-to-noise ratio (SNR) was estimated for fat (subcutaneous), muscle (psoas), and air (preumbilical) in an axial reconstruction with 1 mm slice thickness (soft tissue kernel; Br40) following the approach of Paul et al [22]. In brief, a constant region of interest was identified and set to 20 mm diameter, density as well as accompanied standard deviation was measured in Hounsfield units with a commercially available DICOM viewer (Visage 7, Visage Imaging Inc.) and divided for resulting signal-to-noise ratio. To exclude possible dose effects on signal-to-noise ratio we also normalized SNR by multiplying the SNR with the inverse square root of matching dose values.

**Table 1:** Likert scales for subjective image evaluation. Scales were provided to the four assessing radiologists before the image evaluation

Image quality		
Score	Diagnostic value	Clinical value
1	Uninterpretable examination	Non-diagnostic
2	Poor image quality	Non-diagnostic
3	Acceptable image quality	Diagnostic
4	Good image quality	Diagnostic
5	Excellent image quality	Diagnostic
Image artifacts		
Score	Artifacts	Clinical value
1	Extremely severe artifacts	Non-diagnostic
2	Major artifacts	Non-diagnostic
3	Minor artifacts	Diagnostic
4	Negligible artifacts	Diagnostic
5	No artifacts	Diagnostic
Image noise		
Score	Noise	Clinical value
1	Extreme noise	Non-diagnostic
2	Major noise	Non-diagnostic
3	Minor noise	Diagnostic
4	Normal noise	Diagnostic
5	No relevant noise	Diagnostic

### DNA double-strand break assessment

The assessment of radiobiological impact in peripheral blood cells was performed in an *ex vivo* phantom study in a similar approach as described elsewhere [15]. Ethical approval for this study was obtained from the responsible ethical review committee of the Medical Association of Rhineland Palatinate, Germany (reference number: 837.084.17(10918)). Written informed consent was obtained from all probands. Peripheral blood samples were collected from four healthy male donors (age 32, 34, 34, and 39 years) in EDTA-Monovettes® (5 ml; Sarstedt) in triplicates with matching sham control samples. Exposure was performed utilizing three sequential standard abdomen protocols (as described above) simulating a protocol including three contrast phases in a PCCT scanner (NAEOTOM Alpha, Siemens Healthineers) and conventional CT (SOMATOM go.Top, Siemens Healthineers) with blood samples placed in an abdominal CT-phantom (oval body phantom, tissue equivalent resin (approx. 35 HU @ 120 kV), QRM) at room temperature. Resulting DLP was 302 and 229 mGy*cm for PCCT and conventional CT, respectively.

The frequency of DNA double-strand breaks (DSBs) within peripheral blood mononuclear cells (PBMCs) was determined by fluorescence microscopic enumeration of colocalizing focal accumulations of the two DSB damage marker proteins γ-H2AX and 53BP1 as described elsewhere [14, 23]. Briefly, blood samples were irradiated and incubated for 20 min at 37 °C. Then, PBMCs were isolated by Ficoll® Paque Plus density centrifugation (Merck). Isolated cells were washed twice with phosphate-buffered saline (PBS), fixed in 70% ethanol, and stored at −20°C. Immunofluorescence staining and DSB foci analysis was performed as described previously [23, 24]. Co-localizing γ-H2AX+53BP1 foci were counted by an experienced investigator (H.S.) in 100 morphologically intact and well-separated cells using a Zeiss Axioimager Z2 epifluorescence microscope equipped with appropriate single and dual-band filter sets. Radiation-induced DSB foci (RIF) were obtained by subtracting the sham values from the respective CT-induced DSB foci numbers for each experiment.

### Statistics

Microsoft Excel (Microsoft Corporation) was used for data handling. Data originating from image evaluation was analyzed for statistical differences using Prism 9 (GraphPad Software). Datasets were tested for normal distribution using the Shapiro-Wilk test. An unpaired t-test was used for parametric data and the Mann-Whitney-U test to evaluate non-parametric data. For DSB evaluation, one-way repeated measures analysis of variance (ANOVA) with Post Hoc test (pairwise t-test with Bonferroni) as well as a two-tailed t-test was performed to test for statistical significance.

## Results

### Study population

Detailed patient characteristics are given in Table 2. Groups did not differ significantly regarding age (*p* = 0.18), diameter (*p* = 0.26), height (*p* = 0.89), weight (*p* = 0.20), and resulting body mass index (*p* = 0.16). Scan length did not differ significantly between groups (*p* = 0.69).

**Table 2:** Patient characteristics of the study population

Patient characteristics		
	Conventional CT	Photon-count CT
Examinations (n)	33	33
Age (years ± SD)	64.96 ± 13.38	59.69 ± 18.08
Male (%)	22 (66.6)	23 (69.70)
Female (%)	11 (33.3)	10 (30.30)
Weight (kg)	81.82 ± 17.84	88.52 ± 23.71
Height (mm ± SD)	1757.19 ± 102.62	1753.55 ± 108.98
Diameter (mm ± SD)	305.85 ± 38.57	316.50 ± 37.44
BMI (± SD)	26.38 ± 4.56	28.21 ± 6.01
Scan length (mm ± SD)	505.44 ± 115.79	495.98 ± 66.32

### General image quality

**Fig. 1:**
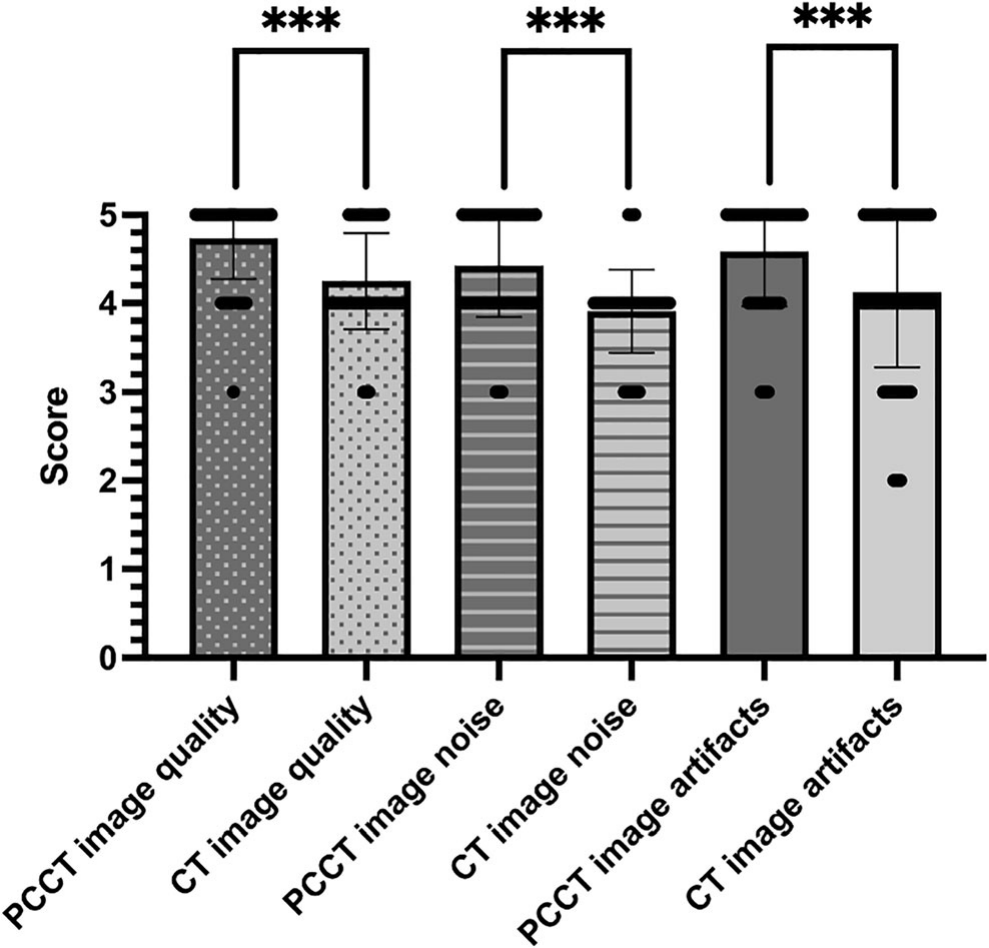
Overall assessment of subjective image properties by four radiologists. Five indicating highest score. (*** *p* < 0.001)

Overall image quality in accordance with the given Likert-scale was found superior for PCCT (4.74 ± 0.46 vs. 4.25 ± 0.54; *p* < 0.001) with scores ranging from 3 to 5 for both modalities (lower 95% CI of mean 4.66 vs. 4.16 and upper 95% CI of mean 4.81 vs. 4.34). In line with this, ratings for image noise (PCCT: 4.42 ± 0.58; conventional CT 3.91 ± 0.47; *p* < 0.001) and image artifacts (PCCT: 4.58 ± 0.62; conventional CT: 4.12 vs. 0.84; *p* < 0.001) showed significantly higher scores for PCCT. Image noise results also ranged from score 3 to 5 (lower 95% CI of mean 4.325 vs. 3.828 and upper 95% CI of mean 4.52 vs. 3.99), whereas scores for image artifacts in conventional CT ranged from 2 to 5 due to six examinations (4.55%) with major artifacts present. Detailed results are depicted in Fig. 1. According to the overall findings, the results of single examiners followed the same trend. For general image quality and image noise, PCCT achieved significantly higher scores (E1 *p* < 0.001 and < 0.001; E2 *p* < 0.001 and *p* < 0.001; E3 *p* = 0.014 and *p* = 0.26; E4 *p* = 0.001 and *p* < 0.001). Also, image artifacts were found to be reduced by each examiner in PCCT (E1 *p* = 0.062; E2 *p* = 0.003; E3 *p* = 0.004; E4 *p* = 0.006). Detailed results are shown in Supplementary Figure 1. The SNR was significantly better for PCCT examinations (*p* < 0.001). For subcutaneous fat, SNR was 6.13 ± 1.72 in PCCT examinations, whereas SNR was 3.39 ± 1.02 in conventional CT. Results of muscle tissue and preumbilical air were similar, ranging from 2.67 ± 0.60 (muscle) to 75.96 ± 19.94 (air) for PCCT and 1.73 ± 0.39 (muscle) to 50.47 ± 21.31 (air) for conventional CT. Both also differed significantly, with *p* < 0.001. After correction for possible dose dependencies SNR still significantly differed for PCCT and conventional CT (muscle *p* < 0.002; fat *p* < 0.001; air *p* < 0.003).

### Dose exposure

Dose exposure was significantly higher for PCCT examinations. Mean DLP was 419.2 ± 162.2 mGy*cm for PCCT vs. 372.3 ± 236.6 mGy*cm for conventional CT (*p* = 0.0435), median DLP was 390 and 315 mGy*cm, respectively. Mean CTDI was 8.38 ± 2.77 mGy vs. 7.09 ± 2.96, (median CTDI was 7.99 and 6.58 mGy*cm) (*p* = 0.0295) for PCCT and conventional CT, respectively. The resulting mean effective dose was calculated (Monte Carlo model–based dose estimation) 5.00 ± 1.95 mSv for PCCT vs. 3.56 ± 2.78 for conventional CT (*p* = 0.0002) (median effective dose was 4.70 and 2.89, respectively). With conversion factor-based calculation, effective dose values were higher but showed the same trend with a mean effective dose of 6.23 (median 5.85) ± 2.43 mSv for PCCT and 5.58 (median 4.73) ± 3.55 mSv for conventional CT. The dose-reference value is determined as 75% quantile of all corresponding examinations in Germany and was last updated in June 2016. For abdominal examinations as described here, the reference value is 15 mGy CTDI_w_body_ and 700 mGy*cm DLP [10].

### Biological impact of dose exposure

**Fig. 2:**
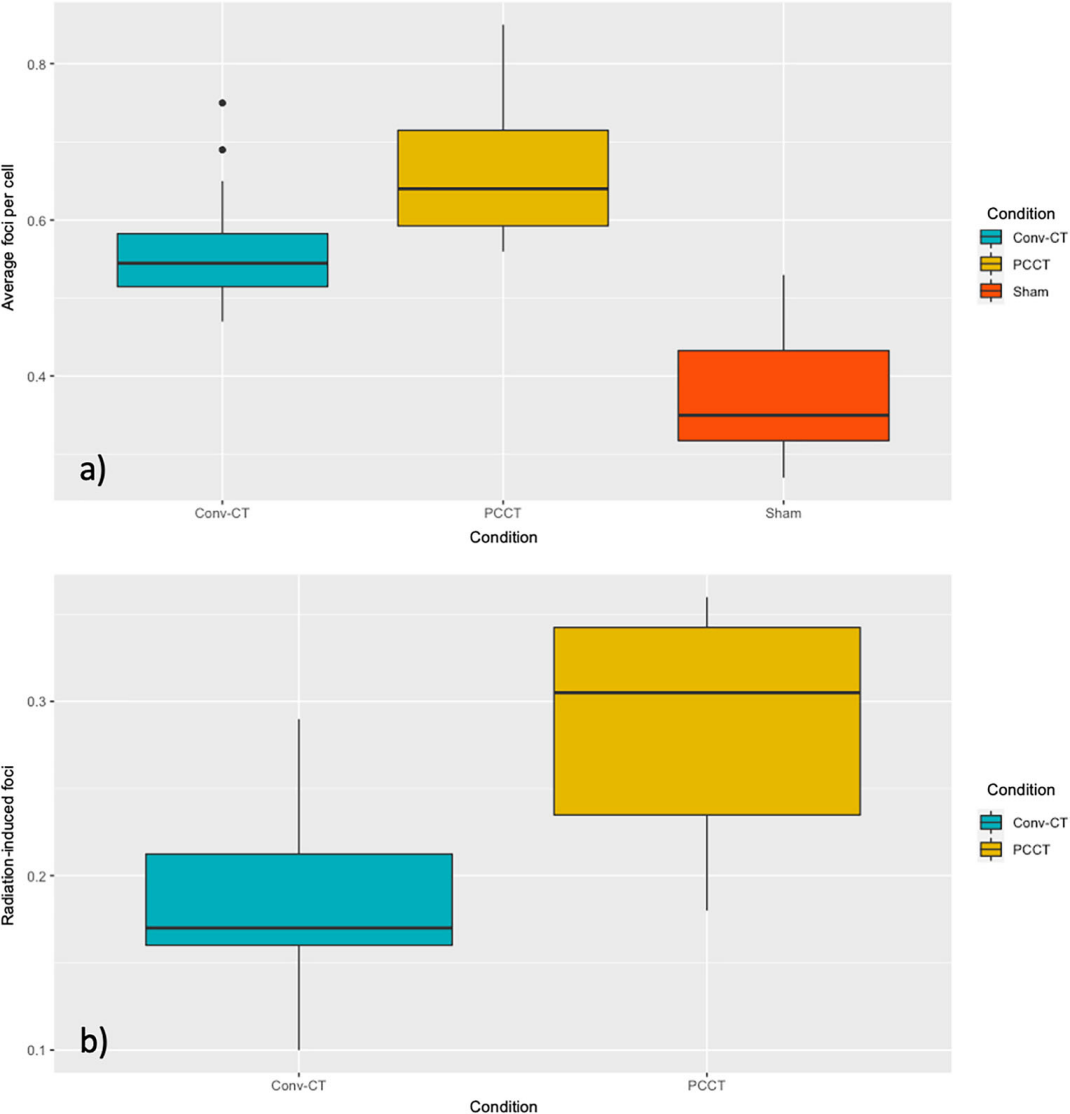
Number of average DNA double-strand break foci per cell (**a**) and radiation-induced foci (**b**) counted after photon-counting CT (PCCT), conventional CT (Conv-CT), and sham exposure (Sham). Boxes display the interquartile range with the median as a horizontal line and whiskers for minimum and maximum. PCCT resulted in significantly elevated average foci numbers per cell (*p* < 0.004) as well as radiation-induced foci (*p* < 0.0001) compared to conventional CT

γ-H2AX+53BP1 colocalizing DSB foci were counted in PBMCs of four individuals after CT- and sham-irradiation in three independent experiments per proband. The mean of the average background DSB foci per cell (FPC) of non-irradiated samples was 0.38 ± 0.073 (FPC ± SD). CT exposure resulted in a highly significant (*p* < 0.0001) increase of FPC in all four individuals with an average DSB foci number of 0.66 ± 0.08 for PCCT and 0.56 ± 0.067 for conventional CT (Fig. 2), in addition to a significant difference among both CT techniques (*p* < 0.004). This finding was mirrored when determining radiation-induced foci (RIF) by subtracting the sham values from the DSB foci values after irradiation. Average RIF numbers for PCCT were significantly (*p* < 0.0001) elevated above the RIF values of conventional CT, 0.28 ± 0.067 vs. 0.18 ± 0.048, respectively. Both differences reflect the elevated DLP of the PCCT.

### First impressions in clinical routine

Some other aspects were not scientifically evaluated but might be of interest. A long training phase did not hamper the implementation of clinical workflows for technicians because of the similar user interface of the PCCT scanner in comparison to the other scanners used in the clinical routine. This could be different when switching from a different manufacturer or user interface. Also, image interpretation needed no major adjustments, as images were reconstructed identically for all scanners and were examined in a standard PACS viewer. Nevertheless, reconstruction and reading of spectral information were not performed for standard clinical examinations and presumably would need further training. Before the operational phase of the PCCT scanner in clinical routine, issues regarding continuous power supply were followed by long (approx. 8 h) downtimes due to a required internal calibration of the detector, which could pose a disadvantage in comparison to conventional detectors.

## Discussion

**Fig. 3:**
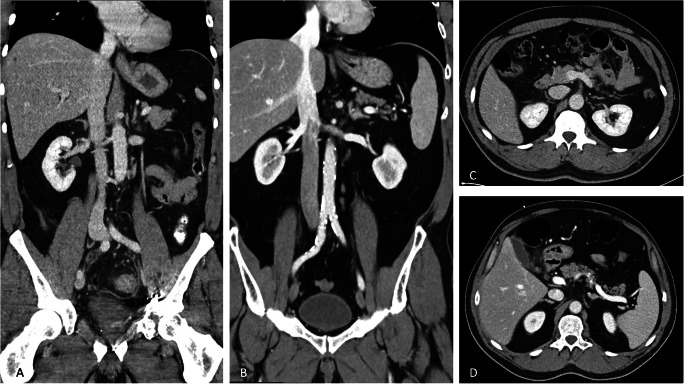
Example of axial and coronal reconstruction of abdominal conventional CT (**A** and **C**) and PCCT (**B** and **D**) for the depiction of subjective image impression. DLP was 413.38 and 453.98 mGy*cm for PCCT and conventional CT, respectively. Both examinations were rated “very good” by all examiners (general image quality PCCT: 4.75 ± 0.5; general image quality conventional CT: 4.75 ± 0.5)

First impressions of abdominal examinations with a novel PCCT scanner in clinical routine were very promising. Overall image quality, image noise, and image artifacts were superior in PCCT compared to conventional CT. A representative example is given in Fig. 3.

In direct comparison, PCCT showed an increase in general image quality, which is consistent with results from previous studies by others comparing PCCT and conventional CT in the evaluation of bony structures [25] or cochlear implants [26]. However, images of conventional CT were also rated “very good” in this study. This possibly reflects the fully completed implementation of a state-of-the-art conventional CT with adapted protocols for abdominal diagnostics based on application experience within the institutional clinical routine. In contrast, protocols used with the PCCT scanner strictly followed the manufacturer’s default settings. This may leave room for further improvement of PCCT by adapting examination protocols based on user experience and future research studies to find an optimal compromise of dose exposure and general image quality for individual clinical cases. The question remains whether a further increase in image quality will lead to a more precise interpretation and, therefore, would directly impact patient care. In most cases, additional information originating from spectral images may not be required. However, the ability to reconstruct spectral images on demand enables radiologists to access new diagnostic capabilities, such as material-specific methods for iodine imaging, energy-specific methods with virtual non-contrast images for evaluating an abdominal mass or artifact reduction in case of beam-hardening as already shown for dual-energy-CT [27]. This may not only strengthen the diagnostic value of CT diagnostics but also prevent (re-) examinations in the further course of clinical care.

Dose exposure of PCCT for abdominal imaging was unsatisfying within the first clinical examinations. Although the mean DLP of PCCT was 40.1% below German dose reference levels for abdominal/pelvic CT [28], the applied radiation dose by an up-to-date conventional CT was even lower (46.9%) in clinical routine. Nevertheless, a dose reduction potential of up to 85% without impairment of image quality has been reported when using PCCT [25]. A possible explanation for this divergence might be the manufacturer's rather conservative protocol recommendation within the early clinical application phase focusing on optimal image quality. In addition, in order to achieve a full spectral data set, the kilovoltage peak is consistently higher at PCCT in comparison to conventional CT (120 kV vs. 80 kV), leading to a higher penetration rate and different beam spectra. Differences between patient groups, such as a slightly greater patient diameter for PCCT patients in this study, also impact the extent of dose exposure. Considering the excellent general image quality of PCCT, a shift towards improved dose reduction seems possible and after patient evaluation for this study mAs values for standard single-phase abdominal protocols have already been reduced within our department. Future investigations including different protocols with an extended and ideally identical patient collective are needed to explore the full potential of PCCT in regard to radiation exposure reduction.

Despite the use of the latest CT generations and device technologies, DNA double-strand break damage was still evident for both scanning techniques, which is consistent with previous observations on modern CT diagnostics [15, 29]. The observed higher average DSB foci per cell values obtained for PCCT agree with the higher DLP of that technique. Given the collected dose values, our results suggest dose-dependent effects of CT irradiation on DNA integrity irrespective of the applied device technology. Influences of device-specific properties, e.g., varying X-ray spectra [30], presumably have a minor impact; however, this assumption needs to be verified by further comparative studies. In this context, it would also be interesting to see how the extent of DNA damage is affected by a comparatively lower amount of contrast agent required in abdominal contrast-enhanced CT due to better iodine contrast-to-noise ratios (CNRs) of PCCT [31].

The study at hand comes with further limitations. Besides the retrospective single-center design, only a few probands were included, and emphasis on abdominal, single-phase CT may not be transposable to other examinations. Also slightly, but not significantly, differing patient characteristics, e.g., patient diameter, could be a confounder and need to be addressed in future investigations.

In conclusion, PCCT examinations offer better general image quality with less image noise and minor image artifacts in abdominal computed tomography in clinical routine. However, dose exposure with standard protocols, as recommended by the manufacturer, is higher than with advanced protocols of a state-of-the-art conventional scanner. Further optimization of PCCT protocols might be required to unleash the full potential of PCCT in clinical routine with a reasonable compromise of image quality and dose exposure. Nevertheless, for specialized examinations, e.g., evaluation of small anatomical structures, low-dose protocols with image noise issues, or spectral analysis the technical advantages of PCCT seem promising [17, 25].

## Supplementary Information

ESM 1 (DOCX 257 kb)

## Abbreviations

ANOVA: Analysis of variance
Br40/Br60: Body regular 40/60
CI: Confidence interval
CNR: Contrast-to-noise ratio
CTDIw body: Weighted computed tomography dose index
DE: Dual-energy
DICOM: Digital Imaging and Communications in Medicine
DLP: Dose-length product
DNA: Deoxyribonucleic acid
DSB: Double-strand break
FDA: US Food and Drug Administration
FPC: Foci per cell
PACS: Picture Archiving and Communication System
PBMC: Peripheral blood mononuclear cells
PBS: Phosphate-buffered saline
PCCT: Photon-counting CT
RIF: Radiation-induced foci
SNR: Signal-to-noise ratio

## Declarations

### Guarantor

The scientific guarantor of this publication is Benjamin V. Becker.

### Statistics and biometry

One of the authors has significant statistical expertise.

### Methodology

• retrospective

• observational/experimental

• performed at one institution
