# Supplementary material for: Initial experience on abdominal photon-counting computed tomography in clinical routine: general image quality and dose exposure
Source: Eur Radiol. 2022 Dec 8;33(4):2461–8. doi: 10.1007/s00330-022-09278-1 (PMC10017564; doi:10.1007/s00330-022-09278-1)

Supplementary Figure 1: Assessment of subjective image properties individually depicted for each assessing radiologist. Five indicating highest score.


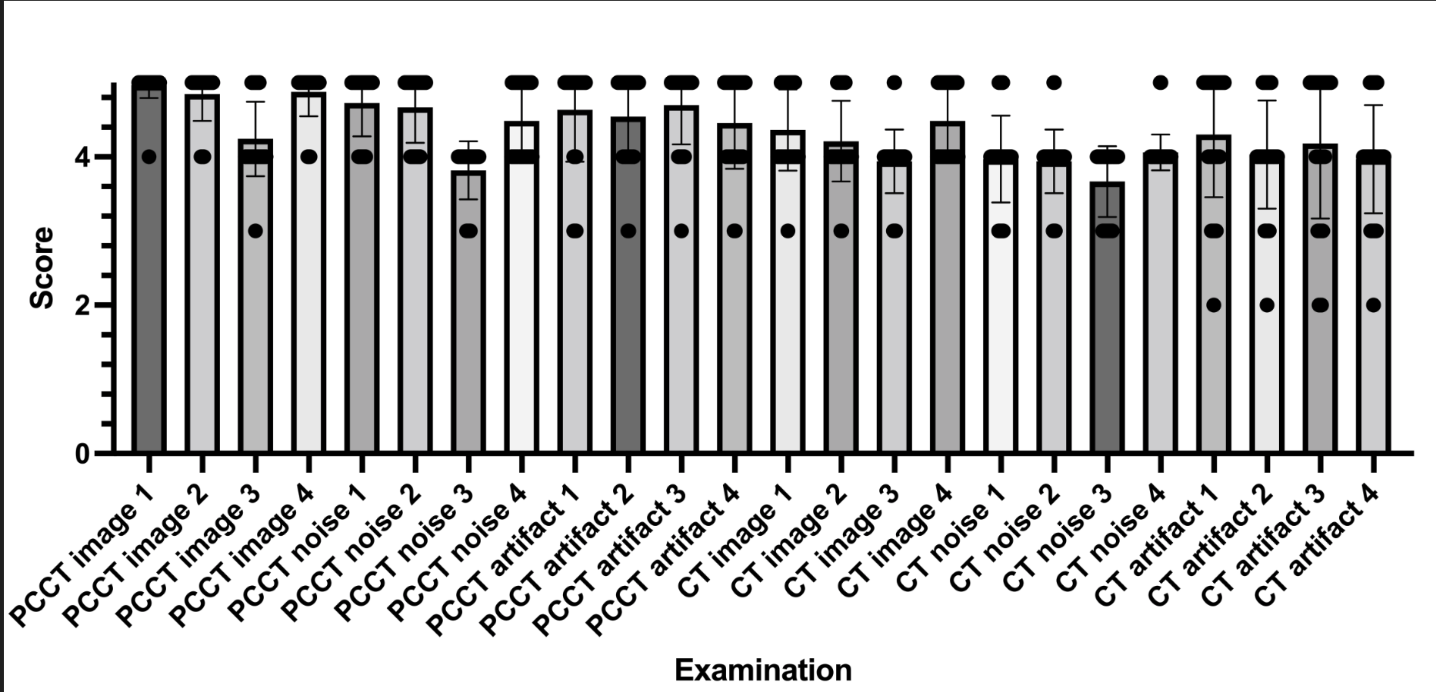

Supplement: Supplementary file 1 — (DOCX 257 kb) [file 330_2022_9278_MOESM1_ESM.docx]
